# Quantitative MRI indicators and features for partial subscapularis tendon tears on conventional shoulder MRI

**DOI:** 10.1186/s13244-022-01307-3

**Published:** 2022-10-20

**Authors:** Qiqi Wang, Jie Zhao, Suying Zhou, Yuchan Lv, Xin Liu, Haitao Yang

**Affiliations:** Department of Radiology, The First Affiliated Hospital of Chongqing Medical University, 1 Youyi Road, Yuzhong District, Chongqing, 400016 People’s Republic of China

**Keywords:** Shoulder, Subscapularis, Tendon tear, Partial tendon tear, Magnetic resonance imaging

## Abstract

**Background:**

Diagnosing partial subscapularis (SSC) tendon tears still faces challenges. A failure rate of massive posterosuperior rotator cuff tear repair will be highly increased when extending more than one-third of SSC tendon. This study aims to investigate the quantitative indicators and features of partial SSC tears on conventional shoulder MRI and improve the preoperative diagnostic accuracy.

**Materials and methods:**

Four hundred and thirty-seven patients underwent MRI and arthroscopy were retrospectively reviewed; 89 patients with partial SSC tears in case group and 50 patients with normal SSC in control group were included. Six MRI features with the explicit definition of some quantitative indicators were evaluated.

**Results:**

Fissure sign showed the highest diagnostic efficiency for the partial SSC tears, with a specificity of 92%, sensitivity of 75.3%, and accuracy of 81.3%. Thinning of SSC, fluid collection under the coraco-glenoid arch (CGA), and combined SSP complete tear also showed high specificity of 86%, 80%, and 80%, respectively, while the sensitivity and accuracy were moderate, with a sensitivity of 38.2%, 50.6%, and 48.3%, respectively, an accuracy of 55.4%, 61.2%, and 59.7%, respectively. The specificity, sensitivity, and accuracy of lesser tuberosity cysts were all moderate with values of 68%, 56.2%, and 60%, respectively. However, fat accumulation under the CGA showed no significant difference between the partial SSC tears group and the control group.

**Conclusion:**

Several specific MRI features with quantitative indicators defined in this study can be used to improve the accuracy of preoperative MRI diagnosis of partial SSC tears.

## Key points

- Quantitative MRI features may improve the accuracy of partial SSC tears.
- Fissure sign shows the highest specificity and accuracy for partial SSC tear.
- Thinning SSC, fluid under CGA, and SSP tears are also meaningful features.

## Background

The subscapularis (SSC) is the largest and most powerful muscle of the rotator cuff [1], and its cross-sectional area is larger than the sum of the other three rotator cuff muscles [2]. It functions as the internal rotator of the shoulder and also plays the role of anterior stabilizer of the humeral head. The tendon of SSC has multiple different planes of insertion to the lesser tuberosity. Its special footprint anatomy makes it difficult to visualize during arthroscopy and some tears of the SSC tendon can be missed, especially the partial tears [3]. The incidence of SSC tears ranges from 27% to 43% in arthroscopy, and partial SSC tears was 19–50% in cadaveric and arthroscopic studies [4–6]. The SSC tear combined with other rotator cuff tears is common, with a reported incidence of 19–49%, and the isolated subscapularis tear is rare, with a prevalence of 4–6% [7–9]. Despite its critical role in the glenohumeral joint, the SSC tendon was long known as “the forgotten tendon”, of which partial tears were thought as the “hidden lesions” [10]. Diagnosing partial subscapularis (SSC) tendon tears still faces challenges. The belly-press test, the most sensitive physical examination test for an upper SSC tear, has only 27.8% sensitivity for identifying partial tears [11–14].

Magnetic resonance imaging (MRI) is currently the most effective and non-invasive tool for preoperative assessment of partial SSC tears, however, the diagnostic accuracy of MRI varies widely in previous studies [15]. A meta-analysis of analyzing the accuracy of MRI in detecting SSC tears concluded that MRI had a sensitivity of 0.74 for partial tears while with 45.4% inconsistency, and specificity for partial tears was 0.88 while with 84.8% inconsistency [16]. Some imaging findings have been investigated to better diagnose SSC lesions, such as leakage of contrast medium beneath the insertion of the SSC, fatty infiltration in the SSC, and abnormalities of the long head of the biceps tendon [17]. However, each of these findings had low sensitivity for detecting tears [18]. Recently, several indirect signs including small defects, fluid collection in superior SSC recess (SSR), and fatty infiltration of the SSC were reported to display high sensitivity [18]. Magnetic resonance arthrography (MRA) has been suggested as an improved diagnostic accuracy method compared with conventional MRI in assessing SSC tendon tears [19, 20]. However, the application of MRA in the evaluation of rotator cuff tears still exists controversial due to its invasive modality and potential complications. The accuracy of preoperative MRA in detecting SSC tendon tears is variable with an overall sensitivity of 31–75%, specificity of 78–100%, and accuracy of 76–89% in diagnosing SSC tendon tears [20–22]. Given the discrepancy of these results, the MRI characteristics of partial SSC tears still need further exploration.

Thus, the objective of this study was to investigate the specific MRI features and quantitative indicators of partial SSC tendon tears by reviewing a large number of partial tear cases and conducting a case–control study based on different imaging manifestations on conventional shoulder MRI.

## Materials and methods

### Subjects

This study was approved by the Committee for Human Research of our institution.

We retrospectively reviewed the clinical records and MR images of 437 patients who received arthroscopic rotator cuff surgery between January 2019 and February 2022 in our institution (Zhao, with 2 years of clinical experience respectively). The inclusion criteria for the case group (partial tears group) were: (1) partial SSC tears diagnosed during shoulder arthroscopy (gold standard); (2) shoulder MRI performed in our institution within 3 months prior to surgery. The exclusion criteria were: (1) Patients with a previous history of shoulder surgery; (2) shoulders with fractures, infections, tumors, autoimmune conditions, or other diseases influencing the SSC evaluation on MRI; and (3) preoperative MR images with poor quality or insufficient sequences for visualizing the SSC.

For the control group (no-tear group), 50 age- and sex-matched cases with intact SSC tendon confirmed in arthroscopy at the same period were selected.

### MRI protocol

All shoulder MRI examinations were performed on a 1.5-T (Magnetom Essenza; Siemens Healthcare) or a 3.0-T (Signa Excite, GE Healthcare) due to multiple MRI scanners in our institution. A dedicated shoulder coil was used with the patient’s arm placed in a neutral position.

The imaging protocol was as follows: intermediate-weighted turbo/fast spin-echo oblique coronal, axial, and oblique sagittal planes as well as T1-weighted turbo/fast spin-echo oblique coronal and sagittal planes. The sequences of MRI scanning included oblique coronal intermediate-weighted fat saturation (IM-WI/fs) (repetition time (TR)/echo time (TE), 2410/44 ms, the field of view (FOV) 17 ∗ 17 cm, slice thickness 3.5 mm), oblique sagittal and axial intermediate-weighted turbo inversion recovery magnitude (TR/TE, 2890–3630/33–37 ms, FOV 18 ∗ 18 cm, slice thickness 4.0 mm), and oblique sagittal as well as oblique coronal T1-weighted turbo spin-echo (TR/TE, 284–346/12–13 ms, FOV 18 ∗ 18 cm, slice thickness 4.0 mm).

### Image analysis

**Fig. 1:**
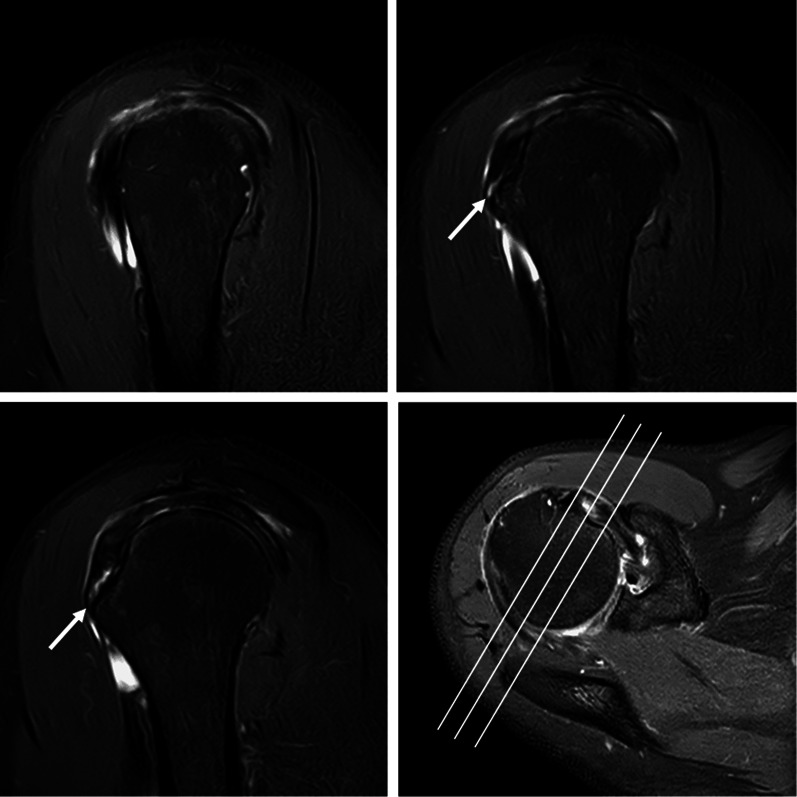
Type-A of fissure sign. There is a transverse fluid-filled gap in the interface between cortex and tendon of SSC footprint at the first three slices on oblique sagittal IM-WI/fs MRI which counted from the most lateral cortex of lesser tuberosity to the medial SSC footprint area referring to the axial MR image, and must be found at least two continuous slices (white arrow)

**Fig. 2:**
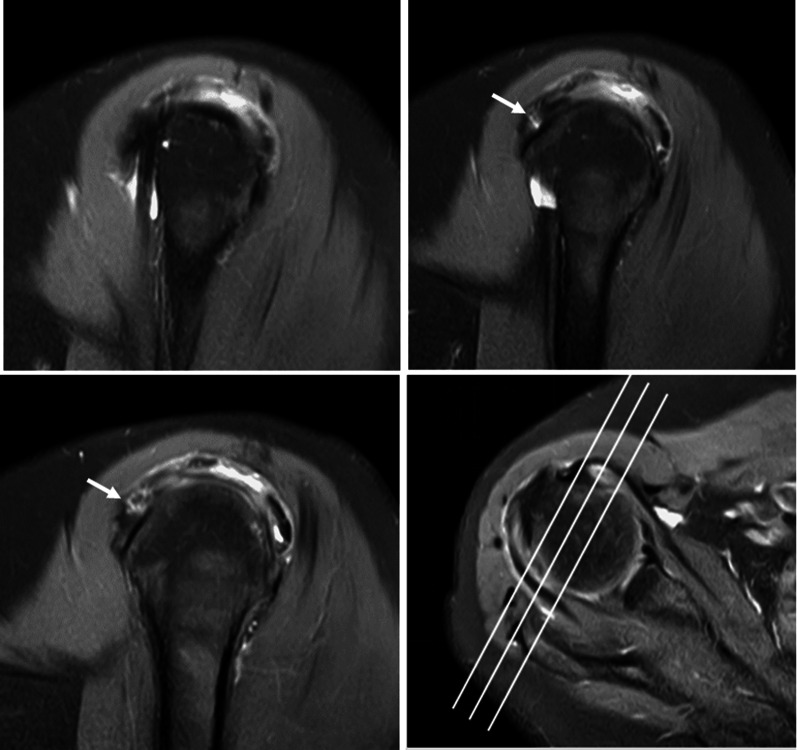
Type-B of fissure sign. There is a vertical fluid-filled gap into the tendon involving both sides surface of SSC at the continuous two slices on oblique sagittal IM-WI/fs MRI counted from the most lateral cortex of lesser tuberosity to the medial SSC footprint area referring to the axial MR image (white arrow)

**Fig. 3:**
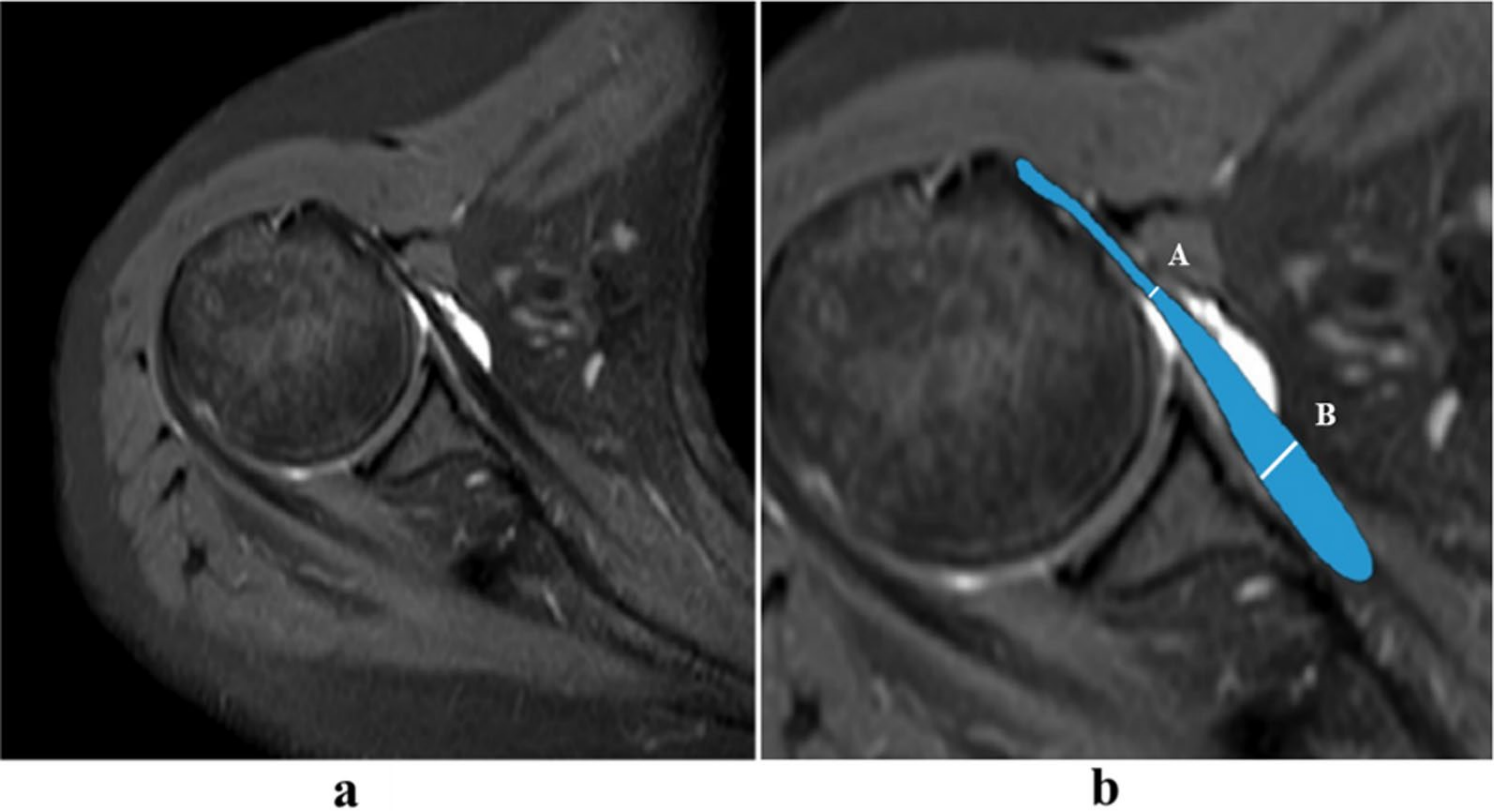
Evaluation of thinning of SSC tendon on axial IM-WI/fs MRI. Manually measuring the diameter of the SSC tendon at its thinnest proximal point (**a**) and its thickest distal point (**b**), and the ratio of a and b is < 1/3 as the reference value of thinning of the SSC tendon

**Fig. 4:**
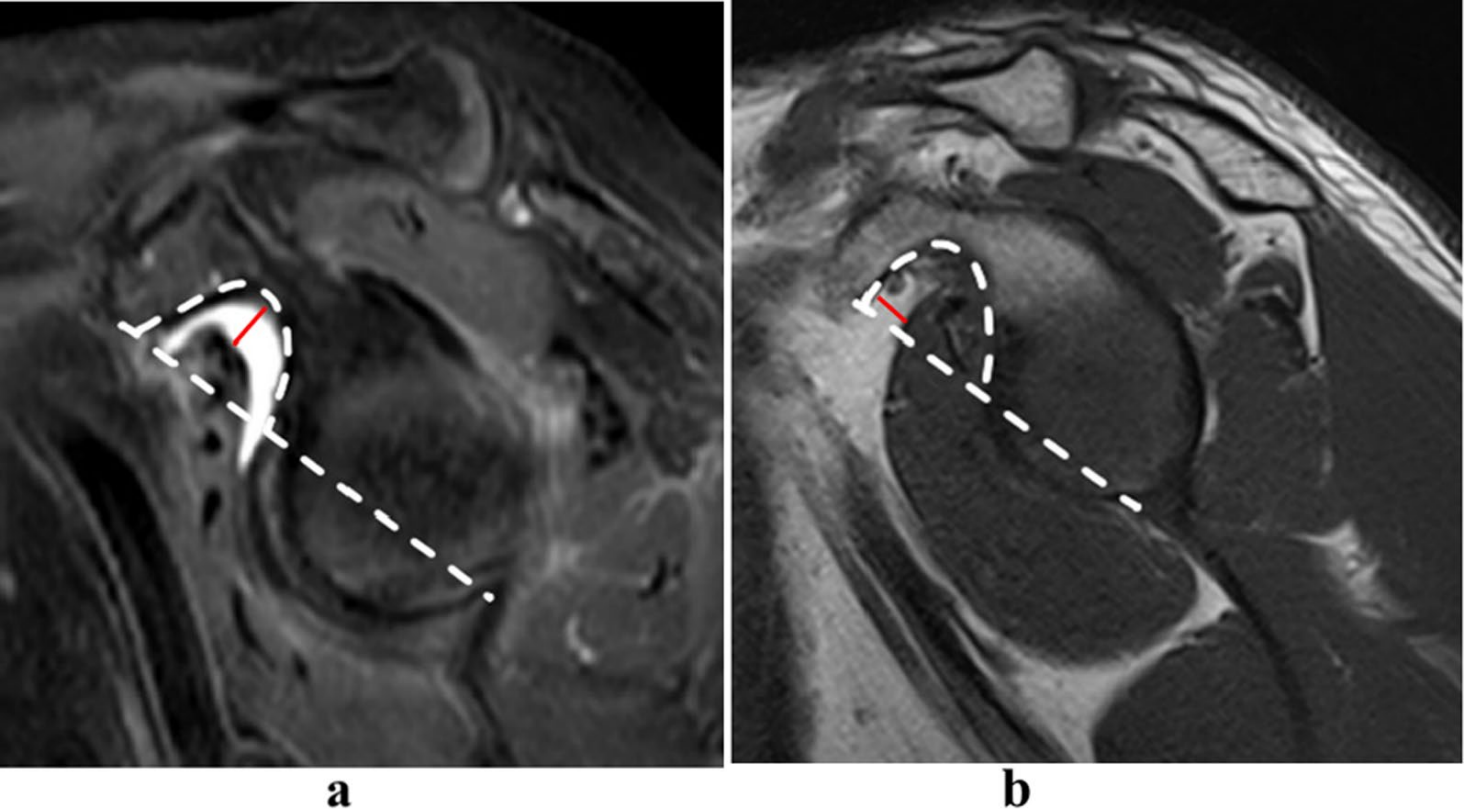
Quantitative measurements of fluid collection and fat accumulation on IM-WI/fs (**a**) and T1-WI (**b**) oblique sagittal MRI at the “coraco-glenoid arch” (CGA) slice. Both the coracoid process and glenoid are well seen and connected by the base of coracoid process on this slice. An arch line can be drawn along the inferior margin of the coracoid and glenoid, another reference line is made connecting the anterior inferior coracoid tip and the leading edge of the insertion of the triceps tendon, and the maximum distance of the fluid or fat accumulated in the coraco-glenoid arch is separately measured

**Fig. 5:**
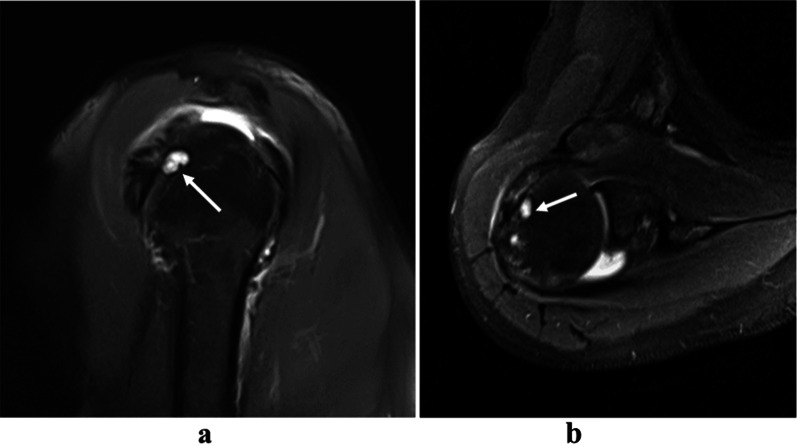
Lesser tuberosity cysts (LTC) on oblique sagittal (**a**) and axial (**b**) IM-WI MRI show well-defined fluid-like cystic lesion within the lesser tuberosity (white arrow)

All MR images were independently reviewed by two musculoskeletal radiologists (Wang and Zhou, with 2 and 3 years of clinical experience, respectively). Six imaging features of MRI findings were subjectively evaluated as follows:

1. Fissure sign. It was defined as a local fluid-filled gap in the footprint of SSC at the first three slices on oblique sagittal IM-WI/fs MRI which counted from the most lateral cortex of lesser tuberosity to the medial SSC footprint area referring to the axial MR image, according to the anatomy of the insertion of the SSC tendon into the lesser tuberosity [3, 17, 18], and must be seen on at least two continuous slices (Figs. 1 and 2). In addition, two subtypes of fissure sign were subsequently divided as follows: type A (Fig. 1), the transverse fluid-filled gap in the interface between cortex and tendon; type B (Fig. 2), the vertical fluid-filled gap into the tendon surface involving both sides of SSC [7, 22, 23].
2. Thinning of the SSC tendon. We manually measured the diameter of the SSC tendon at its thinnest proximal point (A) and its thickest distal point (B) at the axial slice where the cross-sectional areas of the humeral head and SSC tendon were the largest on IM-WI/fs MRI. If the ratio of A and B was < 1/3, we considered it as thinning of the SSC tendon [14] (Fig. 3).
3. The fluid collection in the subcoracoid area. This sign was measured and evaluated at the slices called “coraco-glenoid arch” (CGA) on oblique sagittal T1WI and IM-WI/fs MRI. It was defined as the one or two planes where the whole morphology of both the coracoid process and glenoid connected by the base of coracoid process can be seen [17, 18]. In this view, an arch line was drawn along the inferior margin of the coracoid and glenoid, and another reference line was made connecting the anterior inferior coracoid tip and the anterior edge of the triceps tendon insertion, and the maximum distance of the fluid under the CGA was measured (Fig. 4a).
4. The fat accumulation in the subcoracoid area. The maximum distance of the fat accumulated under the CGA was measured and evaluated at the slices which were similar to define the fluid collection on oblique sagittal T1WI and IM-WI/fs MRI [24] (Fig. 4b).
5. Lesser tuberosity cysts (LTC): The presence of LTC was determined as focal well-defined fluid-like signal intensity lytic lesions within the lesser tuberosity on IM-WI/fs MRI [25], and it must be simultaneously displayed in at least two different cross-sections (Fig. 5).
6. Combined supraspinatus tendon (SSP) complete tears: full-thickness tears of SSP were also evaluated on MRI and confirmed by arthroscopic records. On MRI, the discontinuity of the SSP tendon extended from the articular surface to the bursal surface with fluid-like signal intensity filled was considered a complete tear [19, 26].

All data, both from the case and control groups, were mixed, and then the mixed ID numbers were distributed to the radiologists for independent review. All evaluations and measurements were repeated by one of the radiologists (Wang), and the interval between the first and second reading was 4 weeks.

### Arthroscopy diagnosis

All shoulders underwent a primary arthroscopic rotator cuff surgery by a group of standardized trained senior orthopedists in the joint section of the orthopedic department in our institution. Comprehensive diagnostic arthroscopy was performed through a standard posterior portal. Since a tear less than 10% of the footprint was regarded as a fraying tendon instead of a real tendon tear [18], in the present study, a partial-thickness SSC tear was defined when at least 4 mm of tendon detachment from the footprint was present. Based on previous anatomic studies, this 4 mm length equals approximately 20% of the superior SSC footprint width [3]. Meanwhile, if the footprint coverage was intact but there was a horizontal split lesion inside the tendon, it was also recorded as a partial-thickness tear. According to the intraoperative findings, the SSC tendon was classified as either normal, a partial-thickness tear, or a full-thickness tear. In addition to SSC, other rotator cuff tendons, the subacromial bursa, labrum lesions, and the long head of the biceps tendon were also evaluated and recorded.

### Statistical analysis

Statistical analyses were performed using SPSS software (version 26.0; SPSS). To compare whether there was a difference between the case group and the control group and different scanners for each of the MRI features, t-test was used for continuous variables (e.g., fluid collection or fat accumulation in the coraco-glenoid arch), and receiving-operator characteristic (ROC) curve was calculated for determining the cutoff values, and Chi-square test was used for categorical variables (e.g., fissure sign, thinning of SSC tendon, LTC, combined SSP complete tear).

Intraobserver reliability and interobserver agreement were analyzed by intraclass correlation coefficient (ICC) for continuous variables and Cohen’s Kappa coefficient (*κ* value) for categorical variables. In addition, 95% confidence intervals (CI) were calculated. The level of significance was set at *p* < 0.05. Finally, the sensitivity, specificity, positive predictive value (PPV), negative predictive value (NPV), and accuracy of these features were calculated using arthroscopic findings as to the gold standard.

## Results

### Demographic characteristics

**Fig. 6:**
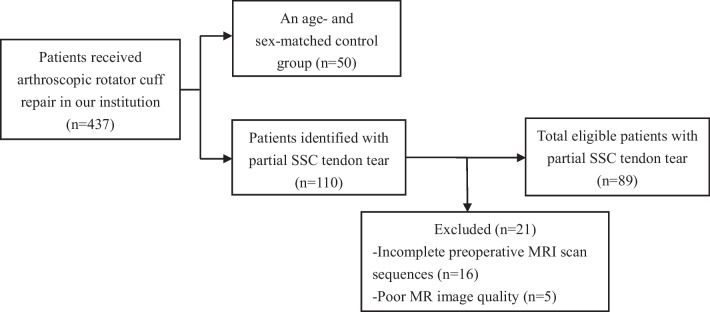
The patient flowchart of the study

Of the 437 patients reviewed in our series, 110 (25%) individuals had partial SSC tears, and 21 patients were excluded from the study for the reasons stated in the flow chart (Fig. 6). Finally, 89 individuals with partial SSC tears were included (34 males, 55 females; age range, 38–91 years; mean age, 61.5 years). The control group included 50 patients (12 males, 38 females, age range 41–77 years, mean age of 58.8 years) with normal SSC observed in arthroscopy. The demographic characteristics of all included patients are presented in Table 1. There were no significant differences in age, sex, and the affected shoulder between the no-tear group (control group) and partial tear group (case group) (*p* > 0.05).

**Table 1:** Comparison of patient demographic description between the case group and control group

	No-tear (control group)	Partial tear (case group)	*p* value
No. of patients	50	89	
Age [mean (range)] (years)	58.8 (range 41–77)	61.5 (range 38–91)	n.s
Sex			
Man	12	34	n.s
Woman	38	55	
Affected shoulder			
Left	15	22	n.s
Right	35	67	

*n.s* Non-significant

### Intra- and interobserver reliability of MRI features and quantitative indicators

The intrareader ICCs for the quantitative measurements ranged from 0.90 to 0.98, whereas the inter-reader ICCs ranged from 0.87 to 0.90. The intrareader *k* value for the categorical variables ranged from 0.82 to 0.84, and the inter-reader *k* value from 0.73 to 0.81, as shown in Table 2, indicating substantial to almost perfect intra- and inter-reader reliability.

**Table 2:** Intraobserver reliability and interobserver agreement for the associated imaging features

Continuous variable	Intraobserver reliability, ICC (95% CI)	Interobserver agreement, ICC (95% CI)
Fluid collection under the CGA	0.90 (0.82–0.94)	0.90 (0.82–0.94)
Fat accumulation under the CGA	0.98 (0.96–0.99)	0.87 (0.80–0.92)

Categorical variables	Intraobserver reliability, *k* value (95% CI)	Interobserver agreement, *k* value (95% CI)
Fissure sign	0.82 (0.65–0.99)	0.81 (0.64–0.99)
Thinning of SSC	0.82 (0.66–0.99)	0.73 (0.54–0.93)
LTC	0.84 (0.69–0.99)	0.80 (0.64–0.97)

Interpretation of ICC value and κ value: values < 0, no agreement; 0–0.20, slight agreement; 0.21–0.40, fair agreement; 0.41–0.60, moderate agreement; 0.61–0.80, substantial agreement; and 0.81–1.00, almost perfect agreement

*SSC* Subscapularis, *LTC* Lesser tuberosity cysts, *CGA* Coraco-glenoid arch, *SSP* Supraspinatus tendon

### Comparison between the partial tear group and the no-tear group

Comparing the quantitative measurements of the fluid collection and fat accumulation under the CGA, there was a significant difference in the fluid collection between the two groups (*p* < 0.05). ROC curve analysis of the fluid collection showed good diagnostic efficacy for partial SSC tears with the area under the curve (AUC) of 0.629 (95% CI 0.543–0.709; Youden’s index, 0.306; cutoff value, 3.55; sensitivity and specificity: 50.6% and 80%, respectively), while no significant difference in fat accumulation under the CGA was found between the two groups (*p* = 0.68).

Five MR imaging features including the fissure sign, thinning of SSC tendon, fluid collection under the CGA, LTC, and combined SSP complete tears were significantly different between the two groups and significantly associated with partial SSC tears (Table 3). Patients with a fissure sign on MRI had the highest risk of partial SSC tears (OR 35.0, 95% CI 11.3–108.4, *p* < 0.001). The risk of partial SSC tears was also increased following other MRI signs, with odds ratios of 2.7–4.8 (95% CI 1.3–9.4, *p* < 0.01).

**Table 3:** Numbers of patients in the control group (no-tear group) and case group (partial tear group) based on MRI findings compared to the gold standard (arthroscopic findings)

MRI findings	Arthroscopy findings		Odd ratio (95%)	*p* value
	No-tear	Partial-tear		
*Fissure sign*				
(+)	4	67	35.0 (11.3–108.4)	< 0.001
(−)	46	22		
*Thinning of SSC*				
(+)	7	34	3.8 (1.5–9.4)	0.003
(−)	43	55		
*Fluid collection under the CGA*				
(+)	10	45	4.1 (1.8–9.2)	< 0.001
(−)	40	44		
*LTC*				
(+)	16	50	2.7 (1.3–5.6)	0.006
(−)	34	39		
*Combined SSP complete tear*				
(+)	10	43	3.7 (1.7–8.4)	0.001
(−)	40	46		

Fluid collection under the CGA: maximum distance of fluid under the CGA > 3.55 mm recorded as (+), otherwise recorded as (−)

*SSC* Subscapularis, *CGA* Coraco-glenoid arch, *LTC* Lesser tuberosity cysts, *SSP* Supraspinatus tendon

The fissure sign showed the highest diagnostic efficiency among these features (sensitivity: 75.3%, specificity: 92%, PPV: 94.4%, NPV: 67.6%, accuracy: 81.3%, respectively). The specificities of the thinning of the SSC tendon, fluid collection under the CGA, and combined SSP complete tears were high (specificity: 86%, 80%, and 80%, respectively), but the sensitivity and accuracy were moderate (sensitivity: 38.2%, 50.6%, and 48.3%; accuracy: 55.4%, 61.2%, and 59.7%, respectively). The specificity, sensitivity, and accuracy of lesser tuberosity cysts were all moderate (68%, 56.2%, and 60%, respectively) (Table 4).

**Table 4:** Diagnostic performance of the associated imaging signs on MRI interpretations compared to arthroscopic findings (gold standard)

	Fissure sign	Thinning of SSC	Fluid collection under the CGA	LTC	Combined SSP complete tear
Sensitivity	75.3	38.2	50.6	56.2	48.3
Specificity	92	86	80	68	80
PPV	94.4	82.9	81.8	75.8	81.1
NPV	67.6	43.9	47.6	46.6	46.5
Accuracy	81.3	55.4	61.2	60	59.7

*SSC* Subscapularis, *CGA* Coraco-glenoid arch, *LTC* Lesser tuberosity cysts, *SSP* Supraspinatus tendon, *PPV* Positive predictive value, *NPV* Negative predictive value

### Comparison of MRI features between the 1.5 T and the 3.0 T scanners

In the case group, there were 66 patients who performed MRI in the 1.5 T scanner, and 23 cases in the 3.0 T scanner, respectively. No significant differences in all MRI features were found between the 1.5 T and the 3.0 T scanners (Table 5) (*p* > 0.05).

**Table 5:** Results of the associated MRI features that were detected by the 1.5 T and 3.0 T scanners in case group (partial tear group)

MRI features	MRI scanners		*p* value
	1.5 T (*n* = 66)	3.0 T (*n* = 23)	
Fissure sign (+)	50 (0.757)	17 (0.739)	0.860
Thinning of SSC (+)	26 (0.394)	8 (0.348)	0.695
Fluid collection under the CGA (+)	32 (0.485)	12 (0.522)	0.761
Lesser tuberosity cysts (+)	36 (0.545)	13 (0.565)	0.870
Combined SSP complete tear (+)	29 (0.439)	14 (0.609)	0.162

Value representation: Frequency (percentage)

*SSC* Subscapularis, *CGA* Coraco-glenoid arch, *SSP* Supraspinatus tendon

## Discussion

In this study, we enrolled a large sample to explore specific imaging features of the partial SSC tears by comparing the case group confirmed by arthroscopy with those of the normal group, verifying that several particular signs and quantitative indicators on conventional MRI can be used to improve the preoperative diagnostic efficacy of partial SSC tears. Compared with the previously proposed predictors, we defined these semi-quantitative MRI signs with more detailed methods of observing and evaluating, aiming to make them more operable and repeatable.

Our study indicated that the “fissure sign” on oblique sagittal MRI was the most specific sign for diagnosing partial SSC tears with high sensitivity (92%) and accuracy (81.3%). In our data, the incidence of the fissure sign was 75.3% (67/89). Small defects and fluid-like signal within the tendon have been reported as a high predictor of partial tears in previous studies [14, 17, 18, 22]. Our results supported this finding, furthermore, the detailed observation approach has been interpreted in this study to objectively evaluate this sign. Most of the partial SSC tears occur in facet 1 of the insertion of the SSC tendon into the lesser tuberosity [17], the width of facet 1 is about 13.8 mm, and has a certain angle of inclination at the tendon insertion [3]. Therefore, we defined the first three slices on oblique sagittal MRI (slice thickness 4.0 mm) counting from the lateral cortex of lesser tuberosity to the medial SSC footprint as the best observation planes, which were approximately equivalent to the width of facet 1 of SSC insertion, and the fluid-filled gap must be found on at least two continuous slices. In our clinical practice, the false-positive fissure sign is more common at the more medial slices to lesser tuberosity, because the tendon already departs the footprint area and joint effusion is easy to flow into the interspace between tendon and humerus head. Subsequently, we continued to define two subtypes of fissure sign according to the orientation of fluid-filled gap, parallel or vertical to the short axis of SSC tendon at footprint corresponding with the tendon detachment and split tear of SSC, respectively [4, 7, 22, 23].

We investigated the fluid collection under the CGA and proposed to establish quantitative measurement methods of them in the slices of the coraco-glenoid arch on oblique sagittal MRI. This area is equal to the part of the SSR which lies between the SSC muscle and the anterior surface of the scapula, extends above the superior margin of the SSC tendon, and communicates with the glenohumeral joint, but not with the subcoracoid bursa [18, 22, 23]. Given the adjacent relation between the SSR and the SSC tendon, partial SSC tears may result in effusions in the SSR due to reactive inflammation or as a result of impingement [27]. Our results showed the fluid collection had a high specificity of 80% and moderate sensitivity of 50.6%. Sugimori et al. [23] reported that SSR fluid had a sensitivity of 100% but a specificity of only 62.5% regarding the incomplete SCC tear detection. We think that our suggested quantification method and the cutoff value (> 3.55 mm) of the effusion may improve its specificity compared to previous studies.

However, no significant difference in quantitative measurement of fat accumulation under the CGA was found between the case group and the control group in our study. Previous studies have shown that muscle atrophy and fatty degeneration can be observed after rotator cuff tendon tears [24]. These are likely the consequence of decreased mechanical load and denervation changes, which results in the proliferation of adipocytes, and ultimately leads to fat accumulation [26]. Several studies evaluated the SSC atrophy or fatty degeneration in SSC lesions [17, 18, 23, 28, 29]. However, SSC atrophy is often seen in a full-thickness tear which frequently represents a significant longstanding of SSC tears. Animal models of SSC tears also showed that complete SSC tenotomy resulted in significant atrophy at 6 weeks, while the changes were not seen in partial SSC tenotomy models [18]. This supported our result that fat accumulation was of no special value to partial SSC tendon tears.

Based on our observations, thinning of the SSC tendon and combined SSP complete tears also showed a good predicting ability of partial SSC tears (specificity: 86% and 80%; accuracy: 55.4% and 59.7%, respectively). Tendon thinning may be due to a reduction of intratendon fibers or a localized defect of the tendon parenchyma [10]. Similar to muscle contraction and tendon lengthening caused by the partial tear of the SSP tendon [30, 31], this may also be the cause of tendon thinning in partial SSC tears. The isolated SSC tears are relatively uncommon. Most SSC tears occur in combination with tears of at least the anterior portion of the SSP [32–34]. Fibers from the SSC and SSP interlock and converge together as they course around and above the humeral head to their respective insertion sites [35], this anatomical structure makes SSC tendon tears usually combined with SSP lesions. Some studies have reported that there is a significant association between SSP full-thickness tear and the presence of SSC abnormality [28, 29], which was consistent with our findings.

The incidence of lesser tuberosity cysts in the partial SSC tears group was 48.3% (43/89) while only 20% (10/50) in the control group in our investigation. And this sign showed only moderate sensitivity (56.2%) and specificity (68%) for partial SSC tears. The relationship between LTC and SSC tears has been reported in several studies [17, 25, 36, 37]. Lee at el. found the lesser tuberosity bone marrow edema and cysts had a sensitivity of 97.8% and specificity of 17.9% in partial SSC tears [17]. As a potential reason for cyst formation, the intact SSC tendon can prevent the lesser tuberosity to contact with synovial fluid after microavulsions, therefore minimizing the fluid-filled cavity anterior to the lesser tuberosity and covering the footprint of the SSC tendon which stays no longer isolated from the synovial fluid after a tear [38]. Chronic impingement of the lesser tuberosity or fluid intrusion through cortical micro-trauma or necrosis may substantially contribute to the formation of the cysts with SSC tears [25, 36]. However, lesser tuberosity cysts are very common in clinical practice. Needell et al. [39] reported that humeral head cysts could also be seen in asymptomatic patients. In addition to be caused by tendon tears, cysts can also develop in other conditions secondary to elevated intra-articular pressure and degenerative changes associated with aging [25].

Finally, all of the MRI features in this study presented a relatively moderate and low sensitivity. We think it may be related to the following reasons: First, to improve the identification and repeatability of these subjective MR features, we gave them relatively clear and rigorous definitions and added some conditional restrictions, so that the positive detection rate of these signs in the case group may be lower than the actual occurrence. Second, although the positive rate of these signs was not very high in the case group, their occurrence was also significantly lower in the normal group, potentially making these signs highly specific in identifying the partial tear and non-partial tear groups.

There are some limitations in this study. First, this is a retrospective study. We only diagnosed the partial SSC tear by referring to arthroscopic records, without accurately matching the MRI features with the intraoperative findings. Second, we did not evaluate the grading and subsection of partial SSC tears due to the limited information of arthroscopic records, and this may potentially decrease the accuracy of these features in certain areas of partial SSC tears. Third, all shoulder arthroscopic surgeries were not performed by one single surgeon, which might result in bias of intraoperative diagnosis. But in our single-center, all the orthopedists were accepted by a standardized course and training, and all the procedures were maintained with the same approach and patient position. Future work is needed to make a prospective control study by comparing preoperative conventional MRI features to arthroscopic findings and develop new imaging techniques such as 3D high-resolution MRI or dynamic MRI sequences in different positions to explore more specific quantification methods for partial tears.

In conclusion, this study identified several specific MRI signs, such as fissure sign, thinning of SSC tendon, and fluid collection under the CGA, with the explicit definition of some quantitative indicators to improve the accuracy of preoperative diagnosis of partial SSC tears on conventional MRI alone.

## Data Availability

The datasets used and/or analyzed during the current study are available from the corresponding author on reasonable request.

## Abbreviations

CGA: Coraco-glenoid arch
ICC: Intraclass correlation coefficient
IM-WI/fs: Intermediate-weighted fat saturation
LTC: Lesser tuberosity cysts
MRI: Magnetic resonance imaging
NPV: Negative predictive value
PPV: Positive predictive value
ROC: Receiving-operator characteristic
SSC: Subscapularis tendon
SSP: Supraspinutus tendon
SSR: Superior subscapularis recess
